# Treatment of Whooping Cough by Atropia

**Published:** 1880-03

**Authors:** Arthur Wiglesworth


### TREATMENT OF WHOOPING COUGH BY ATROPIA.

BY ARTHUR WIGLESWORTH, L. R. C. P.

M. R. C. S., &C.

I was led to try Atropia by reading Trousseau's remarks, concerning the oftentimes beneficial results obtained from belladonna. The alkaloid, however, seemed to be more likely to prove useful; first, because of its unvarying strength; secondly, because the dose could be more easily regulated in consequence; thirdly, because it is nearly tasteless. I therefore chose the solution of the sulphate of atropia (B. P.), which contains 1-120th of a grain in each minim—a most convenient strength; and in every case directed that it should be administered in the morning fasting.

It required some little time to find out the average dose to begin with; but I now begin with 1-120th of a grain (or one minim in a drachm of water) in children from one to four years of age, either diminishing or increasing the dose as occasïon dictates; and, except in very severe cases, only order it to be given once a day, but when the nightly paroxysms are very severe, I order half the dose to be repeated about an hour before bedtime.

The results that follow its administration may be summed up thus: first, a steady diminution in the *number* of paroxysms; second, a diminution in the *duration* of the paroxysms; thirdly, a change in the character of the "whoop," as if the vocal cords were not so closely approximated. Further, if the atropia is withheld, the beneficial effects derived from it, subside.

Now, these results follow more or less speedily the administration of the remedy and appear to depend upon the susceptibility of the patient to the action of the atropia. In a few cases thirst may become a prominent symptom, which subsides, however, on the diminution of the dose. In only one case has the sensation of "falling down" been experienced, and this disappeared with a reduction in quantity.

I append a few cases, taken at random from details before me. In one, the remedy was commenced as soon as the characteristic whoop was definitely established, and the daily number of paroxysms ran up from eleven to twenty-six in four days; by that time the atropia seemed to have begun to act, and in fourteen days the number was reduced to two. In another case the number was twenty-four on the second day after the atropia was commenced and a similar decline took place. Again twenty-six was the number registered; in three days it had fallen to thirteen, remained stationary for a week, then steadily declined. In another patient, aged ten years, the cough was almost incessant: in a week there was no paroxysm to record. One case I should like to give more in detail: The patient, a little girl, aged five, had passed through a severe catarrhal stage, and the "whoop" had become well established, when she had a sharp attack of measles, but no diminution of the cough. The rash was nearly gone, when acute broncho-pneumonia of both lungs set in, dullness on the left side being well marked half-way up the lung, respiration twenty-five in a minute; dyspnœa urgent; recumbent position impossible; pulse 170; fluids could only be taken in single mouthfuls for want of breath. Each pertussal paroxysm caused the greatest distress. In this case, I ordered 1-120th of a grain of sulphate of atropia twice a day with the most satisfactory results. The cough became less frequent, and its duration was decidedly shortened and less violent. She was also ordered in the interval carbonate of ammonia and decoction of senega. Under this treatment, the child slowly rallied and is now perfectly well. A good example recently occurred in a family of a clergyman: three of the children being attacked with whooping-cough, coincidently with their neighbor's family, with whom they played every day. In a month all trace of disease had left them, whilst at the end of two months their neighbor's children still had it.

Whooping cough is essentially a "neurosis," and if we are to judge from the sensations described to us by those who are old enough to analyze their feelings, it is the laryngeal branches of the pneumograstric nerve that are primarily affected.

Of all the drugs, there are none that have such a peculiar and special effect upon the pneumogastric nerve as belladonna, though it is by no means limited to that nerve. It is essentially a nervine sedative, and has a capacity for diminishing both sensibility and irritability when these are morbidly increased. Its primary effects are manifested upon the mouth and throat producing thirst. A further action is upon the laryngeal muscles, rendering articulation imperfect, or preventing it altogether. It is reasonable, then, to attribute the beneficial effects of atropia in whooping cough, chiefly to its effect upon the laryngeal branches of the pneumogastric nerve, diminishing its exalted sensibility and irritation which are known to exist, and which by constant propagation to the medulla oblongata, increase in that body the capacity for reflex phenomena. But it is also probable that atropia has a very decided effect upon the medulla oblongata itself, rendering it less capable of exciting reflex action. Dr. Kroon's experiments led him to the conclusion that valerianate of atropia had a very special and direct effect upon diminishing its inherent capacity for reflex phenomena.

I think then the conclusion is justified that by its action upon the pneumogastric and sympathetic nerves, and also upon the medulla oblongata, atropia relieves and ultimately cures the neurosis, called whooping cough; and that in those cases when, by idiosyncracy or easily-excited sympathetic action, the intensity and severity of the reflex phenomena are greatest, the beneficial action of atropia will be more marked.—*London Lancet, Aug., 1879.*

